# Exosomal ANGPTL2 in cerebrospinal fluid as a novel prognostic biomarker for primary central nervous system lymphoma

**DOI:** 10.3389/fimmu.2026.1699805

**Published:** 2026-01-29

**Authors:** Lili Zhu, Qing Li, Jingjing Ma, Huiwen Xu, Yan Ma, Zhiguang Lin, Mengxue Zhang, Songmei Wang, Xuanyi Wang, Bobin Chen

**Affiliations:** 1 Department of Hematology, Huashan Hospital, Fudan University, Shanghai, China; 2 Laboratory of Medical Molecular Biology, Experimental Teaching Center, School of Basic Medical Sciences, Fudan University, Shanghai, China; 3 Key Laboratory of Medical Molecular Virology of the Ministry of Education and The Ministry of Health, School of Basic Medical Sciences, and Institutes of Biomedical Sciences, Fudan University, Shanghai, China

**Keywords:** ANGPTL2, cerebrospinal fluid, exosomes, primary CNS lymphoma, prognosis

## Abstract

**Background:**

Angiopoietin-like protein 2 (ANGPTL2) functions as a key pro-tumorigenic mediator in the tumor microenvironment. While liquid biopsies using exosomal proteins to reflect tumor phenotypes and progression are well established for extracranial cancers, their use in primary central nervous system lymphoma (PCNSL) remains unexplored. Therefore, this study aimed to assess the potential of exosomal ANGPTL2 in cerebrospinal fluid (CSF) as a prognostic biomarker for PCNSL.

**Methods:**

We retrospectively reviewed the medical records of patients newly diagnosed with PCNSL at our institution from May 2020 to September 2023. Using enzyme-linked immunosorbent assay (ELISA), we measured CSF exosomal ANGPTL2 levels in 78 patients with PCNSL. Patients were divided into high- and low-level groups based on the median. The Kaplan–Meier analysis with a log-rank test was used to compare survival rates between groups. Univariate analysis was used to identify prognostic factors, and multivariate Cox proportional hazards regression was used to determine the independent predictors of PCNSL outcomes.

**Results:**

The median level of CSF exosomal ANGPTL2 was 4.410 ng/mL. Patients with high CSF exosomal ANGPTL2 had significantly shorter progression-free survival (PFS; *p* = 0.001) and overall survival (OS; *p* = 0.003). The complete response (CR) rate was 33.3% in the high-level group versus 66.7% in the low-level group (*p* = 0.003), and the overall response (OR) rate was 35.9% versus 74.4% (*p* = 0.001).

**Conclusion:**

CSF exosomal ANGPTL2 is a novel, independent prognostic marker in PCNSL.

## 1 Introduction

Primary central nervous system lymphoma (PCNSL), a rare and highly aggressive extranodal non-Hodgkin’s lymphoma (NHL), is strictly confined to the central nervous system (CNS), including the brain parenchyma, spinal cord, cranial nerves, leptomeninges, and vitreoretinal structures, without systemic involvement (1, 2). Despite improvements in chemotherapy and emerging immunotherapies, PCNSL remains associated with poor prognosis, with a median overall survival (OS) of less than 2 years and a 5-year survival rate below 40% (3, 4). Relapse occurs frequently, and treatment options for refractory disease are limited, highlighting the urgent need for tools to guide risk stratification and optimize clinical management (5).

In addition to therapeutic challenges, accurately assessing disease in its early stages is difficult. Stereotactic biopsy remains the gold standard for diagnosis but carries inherent procedural risks that may affect patient compliance (6). Auxiliary cerebrospinal fluid (CSF) diagnostics (e.g., flow cytometry) are further limited by low lymphocyte yields and low detection sensitivity (7). Moreover, the blood–brain barrier (BBB) restricts the use of serum-based biomarkers (8). These challenges underscore the urgent need for minimally invasive prognostic tools to guide risk stratification and clinical decision-making in PCNSL patients.

Exosomes, nanoscale extracellular vesicles 30–150 nm in diameter, are secreted by virtually all cell types and carry proteins, nucleic acids, and lipids that reflect the physiological or pathological state of their cells of origin (9). Their stability in biological fluids and protection of cargo from enzymatic degradation make exosomal proteins attractive candidates for non-invasive biomarkers, particularly in CNS malignancies, where tissue access is limited and serum-based markers are often unreliable (10, 11). Leveraging this concept, our previous proteomics study revealed significantly elevated levels of angiopoietin-like protein 2 (ANGPTL2) in CSF exosomes from PCNSL patients compared with those from individuals with benign CNS disorders, suggesting active secretion and potential biological relevance.

ANGPTL2 is a secreted glycoprotein structurally related to angiopoietins, which regulate vascular homeostasis and angiogenesis (12). In addition to its physiological roles, ANGPTL2 contributes to tumorigenesis in multiple malignancies through chronic inflammatory signaling, extracellular matrix remodeling, promotion of angiogenesis, and establishment of an immunosuppressive microenvironment (13–16). Elevated ANGPTL2 expression has been linked to aggressive tumor behavior, metastasis, and poor prognosis in both solid and hematologic cancers (17–19). Given the immune-privileged environment of the CNS, these pathological effects may be particularly relevant to PCNSL, in which vascular abnormalities and immune evasion play central roles in disease progression.

Despite these insights, the prognostic value of exosomal ANGPTL2 in CSF remains unexplored. Considering the limitations of current diagnostic and prognostic strategies in PCNSL, the stability and tumor-reflective nature of exosomal proteins, and our prior observation of elevated CSF exosomal ANGPTL2, investigating its clinical relevance is highly warranted. Therefore, this study aims to evaluate CSF exosomal ANGPTL2 as a novel prognostic biomarker by correlating its expression with survival outcomes and clinical features in patients with PCNSL, potentially informing risk stratification and therapeutic decision-making.

## 2 Materials and methods

### 2.1 CSF samples and patient clinicopathological data

In this retrospective study, CSF samples were collected from 78 treatment-naive patients with newly diagnosed PCNSL at our institution between May 2020 and September 2023, who had available pretreatment CSF samples and complete follow-up information. All samples were obtained before any antitumor therapy. Patients met the following inclusion criteria: (1) initial CNS or ocular symptoms without systemic lesions; (2) absence of lymphoid/hematopoietic tissue or extraneural organ involvement, confirmed by comprehensive physical examination and auxiliary diagnostics; and (3) newly diagnosed, untreated PCNSL. Pretreatment imaging and histopathological confirmation were required for enrollment. Treatment response was assessed by comparing post-chemotherapy imaging data with baseline data. Follow-up was censored on 31 May 2025. Primary endpoints were progression-free survival (PFS; defined as time from diagnosis to radiographically confirmed progression or death) and OS (defined as time from diagnosis to death from any cause). All participants received high-dose methotrexate (HD-MTX)-based chemotherapy regimens.

### 2.2 Isolation and characterization of exosomes

The CSF samples underwent an initial centrifugation for 10 min to remove cells, followed by a second centrifugation at 2,500 × g for 10 min to eliminate cell debris, apoptotic bodies, and larger particles. The supernatant was collected and stored at −80°C for subsequent analysis. All samples were processed using the same preanalytical workflow to minimize variability. For each subject, exosomes were isolated from a fixed input volume of CSF (1 mL) to ensure volume-based normalization across samples.

For exosome isolation, the collected CSF was centrifuged (Eppendorf AG, Germany) at 2,500 × g for 10 min at 4°C to remove cell debris and then filtered through a 0.22-μm filter (Millipore, MA, USA). The supernatant was mixed with ExoQuick-TC™ exosome precipitation solution (System Biosciences, Palo Alto, CA, USA, Cat. #EXOTC10A-1) at a 5:1 ratio (v/v) and incubated overnight at 4°C. The mixture was subsequently centrifuged at 10,000 × g for 1 h at 4°C, after which the yellow exosome pellets were harvested. Transmission electron microscopy (TEM; HT7700, Hitachi) and nanoparticle tracking analysis (NTA; NanoSight NS300, Malvern Panalytical) were used to determine the size and shape of the exosomes. The characterization of the exosomes was verified by detecting the expression of the exosome-specific marker TSG101 (1:4,000; Abcam, Cambridge, MA, USA, Cat. #ab125011) and the exosome-associated protein marker CD81 (1:4,000; Abcam, Cambridge, MA, USA, Cat. #ab219209) by Western blotting.

### 2.3 Enzyme-linked immunosorbent assay

The cerebrospinal fluid level of exosomal ANGPTL2 was assessed using the Human ANGPTL2 ELISA Kit (Jianglai Biological, China, Cat. #JL19947-96T) according to the manufacturer’s instructions. Briefly, standards and diluted samples were dispensed in duplicate (100 μL/well) and incubated at 37°C for 1 h. After aspiration, 100 μL of biotinylated detection antibody (1×) working solution was added directly to each well without washing, followed by a second 1-h incubation at 37°C. The plate was then aspirated and washed three times with 300 μL of wash buffer per well. Subsequently, 100 μL of enzyme conjugate working solution was added and incubated at 37°C for 30 min. After another five washes, 90 μL of TMB substrate was added to each well and incubated in the dark at 37°C for 15 min. The reaction was terminated with 50 μL of stop solution, and the optical density was immediately detected at 450 nm using an xMark™ Microplate Absorbance Spectrophotometer (Bio-Rad, USA, Cat. #1681150). The analytical detection limit of the assay, as specified by the manufacturer, ranged from 0.31 to 20 ng/mL.

### 2.4 Statistical analysis

Statistical analyses were conducted using GraphPad Prism (version 9.0, GraphPad Software, San Diego, CA) and SPSS Statistics 20(IBM, Armonk, NY). Continuous variables are presented as mean ± standard deviation (SD) or as median with interquartile range (IQR), depending on distribution normality, assessed by the Shapiro–Wilk test. Categorical variables are expressed as frequencies and percentages.

Intergroup differences in ANGPTL2 levels were analyzed using Student’s t-test (parametric) or Mann–Whitney U test (non-parametric), as appropriate. Kaplan–Meier survival curves were generated to evaluate the association of exosomal ANGPTL2 expression with clinical outcomes, and between-group differences were assessed using the log-rank test. Prognostic factors were identified using univariate Cox proportional hazards regression. Variables that were statistically significant in univariate analysis were included in multivariate Cox regression models. Statistical significance was defined as two-sided *p* < 0.05.

## 3 Results

### 3.1 Clinical characteristics of the included PCNSL patients

**Figure 1:**
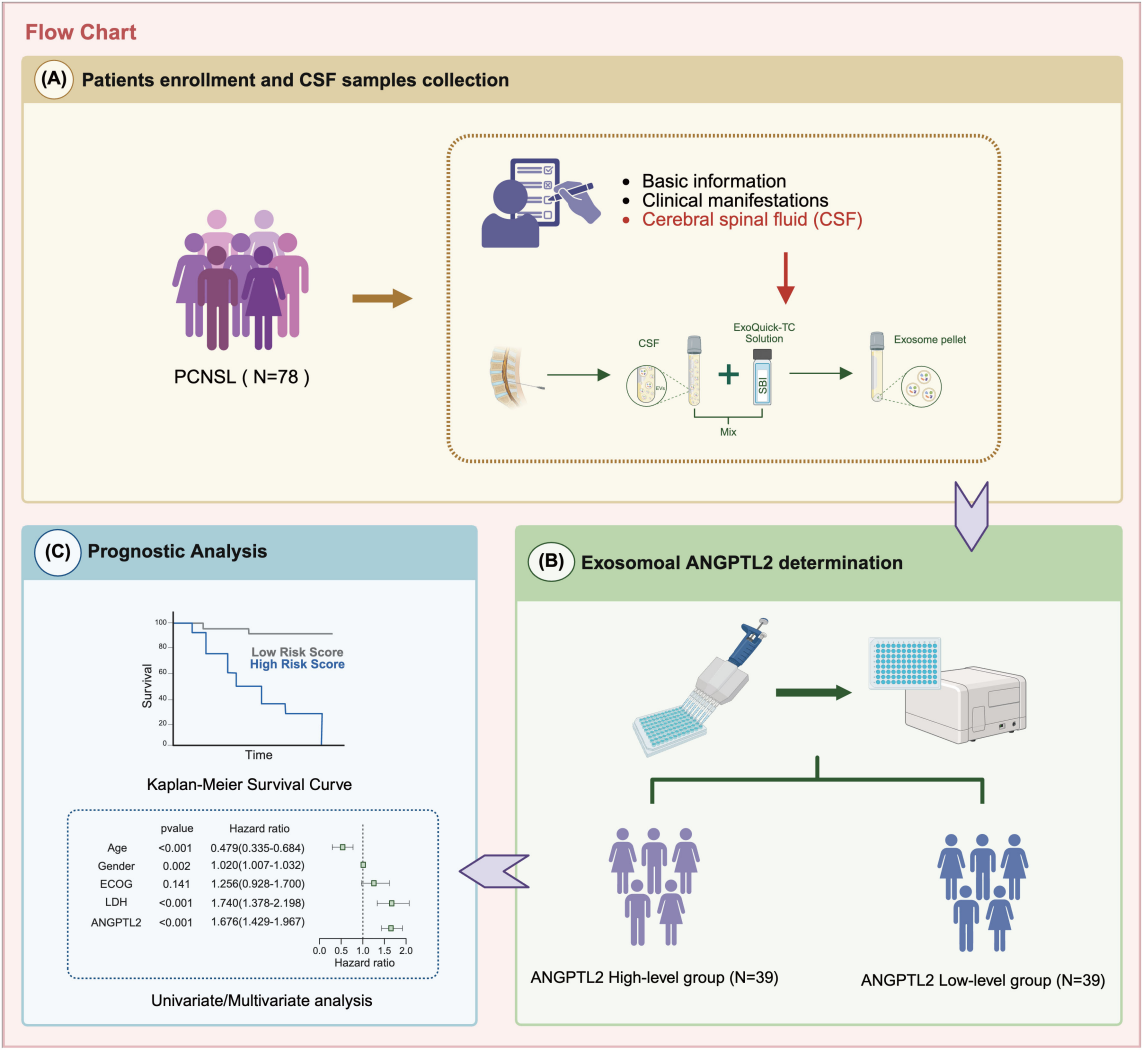
The study flowchart. Patients were divided into ANGPTL2 high- and low-level groups based on exosomal ANGPTL2 levels in CSF. Prognostic analysis was used to evaluate its value for PCNSL.

The retrospective cohort enrolled 78 consecutive PCNSL patients who fulfilled the 2022 WHO diagnostic criteria at Huashan Hospital between May 2020 and September 2023. The overall study design is illustrated in **Figure 1**. The cohort had a median age of 60 years (range, 28–78 years), with a male predominance (male-to-female ratio 1.6:1). Comprehensive baseline characteristics, including Eastern Cooperative Oncology Group (ECOG) performance status, serum lactate dehydrogenase (LDH) elevation, lesion localization patterns, and biopsy type, are comprehensively detailed in **Table 1**.

**Table 1:** Clinical characteristics of the included PCNSL patients.

Characteristics	Patients (n=78)
Age, n (%), years	
> 60	38(48.72%)
≤ 60	40(51.28%)
Median age (range)	60[28-78]
Sex, n (%)	
Male	48(61.54%)
Female	30(38.46%)
ECOG, n (%)	
0-1	43(55.13%)
2-4	35(44.87%)
LDH, n (%)	
Elevated	4(5.13%)
Normal	74(94.87%)
No. of lesions, n (%)	
1	45(57.69%)
≥ 2	33(42.30%)
Deep brain lesions, n (%)	
No	23(29.49%)
Yes	55(70.51%)
Biopsy type	
Surgical	24(30.77%)
Stereotactic	54(69.23%)

ECOG, Eastern Cooperative Oncology Group; LDH, lactate dehydrogenase.

### 3.2 Morphological and biochemical characterization of the extracellular vesicles prepared from CSF of PCNSL patients

Several methods are currently available for isolating exosomes, differing in yield and quality. In this study, we employed a commercial exosome isolation kit as a standard operating procedure (SOP) to isolate exosomes from CSF. The obtained exosomes were characterized by TEM, NTA, and Western blotting for canonical exosomal markers.

**Figure 2:**
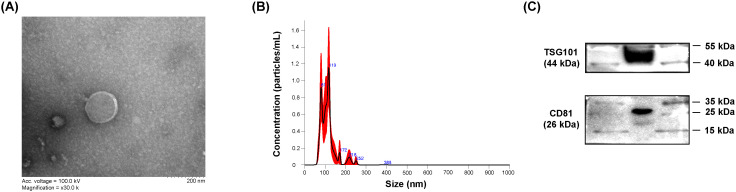
Characterization of CSF-derived exosomes. **(A)** TEM image showing the characteristic spherical morphology of exosomes. Scale bar, 200 nm. **(B)** NTA profile depicting the size distribution of exosomes, with a mean diameter of 118 nm. **(C)** Western blot analysis confirming the presence of exosomal markers TSG101 (44 kDa) and CD81 (26 kDa).

TEM analysis revealed that the CSF-derived exosomes exhibited a characteristic spherical morphology with intact lipid bilayer membranes (**Figure 2A**). NTA showed that the exosome size distribution ranged from 30 to 150 nm, with the main peaks between 80 and 120 nm, confirming that the majority of particles fall within the expected exosome size window (**Figure 2B**). Western blotting confirmed the presence of canonical exosomal markers TSG101 and CD81, detected at 44 kDa and 26 kDa, respectively (**Figure 2C**). Collectively, these findings confirm that the isolated vesicles meet the consensus criteria for exosomes and are suitable for subsequent biomarker analyses.

### 3.3 Determination of exosomal ANGPTL2 expression in PCNSL patients

**Figure 3:**
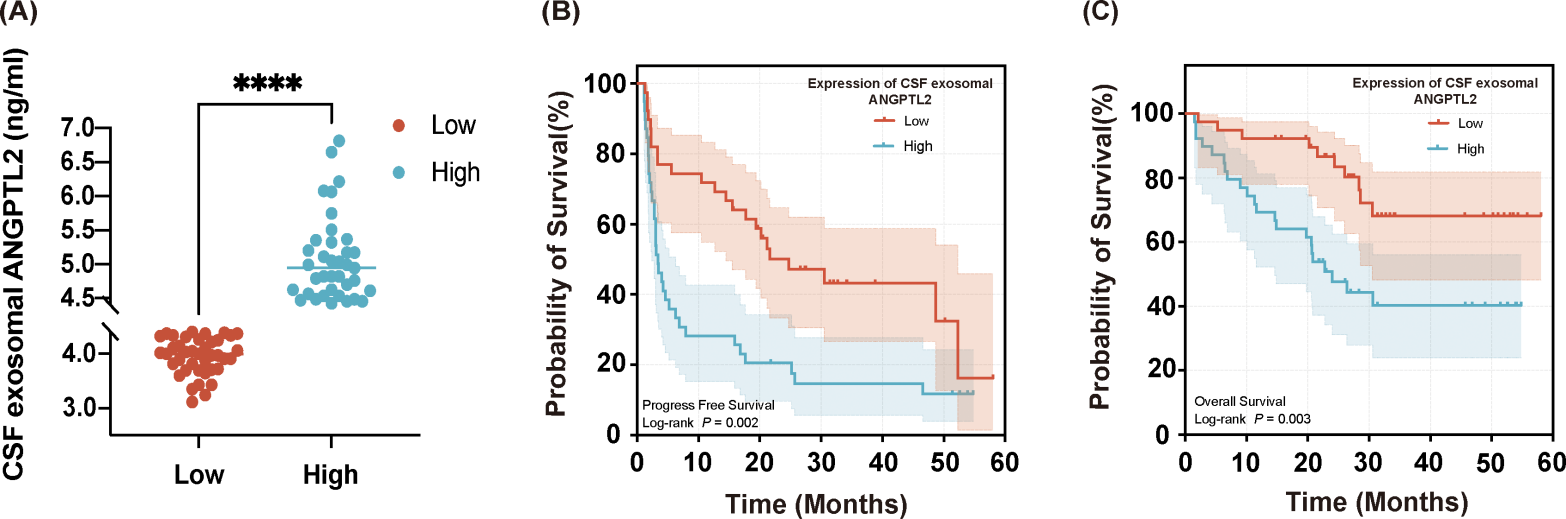
Kaplan–Meier curves for PCNSL patients stratified by CSF exosomal ANGPTL2 levels. **(A)** Distribution of CSF exosomal ANGPTL2 in the high-level (≥ median) and low-level (< median) groups. ****, p < 0.0001. **(B)** PFS curves for patients with high versus low exosomal ANGPTL2 levels. *p = 0.002*. **(C)** OS curves for patients with high versus low exosomal ANGPTL2 levels. *p = 0.003*.

Exosomal ANGPTL2 levels in CSF were quantified using enzyme-linked immunosorbent assay (ELISA). The median exosomal ANGPTL2 concentration was 4.410 ng/mL (range: 3.114-10.190 ng/mL). Patients were stratified into two groups based on the median ANGPTL2 level: a high-level group and a low-level group. Exosomal ANGPTL2 levels were significantly higher in the high-level group than in the low-level group (*p* < 0.0001; **Figure 3A**). The baseline characteristics of patients in the high- and low-ANGPTL2-level groups are presented in **Table 2**. No significant differences in baseline characteristics were observed between the high- and low-level ANGPTL2 groups.

**Table 2:** Comparison of baseline characteristics between the ANGPTL2 high- and low-level groups.

Characteristics	ANGPTL2 high-level group (N = 39)	ANGPTL2 low-level group(N = 39)	*p* value
Gender, n (%)			0.351
Male	22(56.41%)	26(66.67%)	
Female	17(43.59%)	13(33.33%)	
Age, median [IQR]	56[49-69]	62[52-65]	0.415
ECOG score			0.111
0-1	18	25	
≥ 2	21	14	
Multiple lesions	19	13	0.167
Involvement of deep structure	29	26	0.456
Biopsy type			> 0.999
Surgical	12	12	
Stereotactic	27	27	
Elevated serum LDH level	2	2	> 0.999
Elevated CSF WBC count	5/35	5/36	0.961
Elevated CSF protein level	20/35	21/36	0.919

IQR, interquartile range; ECOG, Eastern Cooperative Oncology Group; LDH, lactate dehydrogenase; CSF, cerebrospinal fluid; WBC, white blood cell.

### 3.4 Association between ANGPTL2 expression and patient survival

Kaplan–Meier survival analysis showed that patients with high exosomal ANGPTL2 levels had significantly shorter PFS and OS than those with low levels (*p* = 0.002 and *p* = 0.003, respectively; **Figures 3B, C**). Prognostic stratification based on exosomal ANGPTL2 expression revealed significant disparities in clinical outcomes (**Table 3**). Patients in the high ANGPTL2 group had significantly lower complete response (CR) rate (*p* = 0.003) and overall response (OR) rate (*p* = 0.001) than the low-expression group, with 2-fold and 2.1-fold reductions, respectively. The median PFS was 3.4 months in the high ANGPTL2 group versus 24.7 months in the low ANGPTL2 group. The median OS was 28.4 months in the high-level group, whereas it was not reached in the low-level group during follow-up.

**Table 3:** Outcomes of the ANGPTL2 high- and low-level groups.

Outcomes	ANGPTL2 high-level group (N = 39)	ANGPTL2 low-level group (N = 39)	χ^2^	*p* value
CR (%)	13(33.33%)	26(66.67%)	8.67	0.003
OR (%)	14(35.9%)	29(74.36%)	11.66	0.001
Median PFS (month)	3.5	24.7	8.682	0.003
Median OS (month)	28.4	NR	9.738	0.002

HD-MTX, high-dose methotrexate; CR, complete response; OR, overall response (complete response + partial response); PFS, progression-free survival; OS, overall survival.

### 3.5 High CSF exosomal ANGPTL2 levels were independently associated with poor prognosis among patients with PCNSL

To identify independent prognostic factors, we performed comprehensive univariate and multivariate Cox regression analyses incorporating clinicopathological and molecular features. In the PFS analysis (**Table 4**), univariate analysis identified several significant prognostic factors, including ECOG performance status, deep brain involvement, number of lesions, CSF protein levels, and elevated exosomal ANGPTL2 levels. Subsequent multivariate analysis identified age (*p* = 0.011), number of lesions (*p* = 0.037), and high exosomal ANGPTL2 levels (*p* < 0.0001) as independent predictors of reduced PFS. Similarly, for OS (**Table 5**), univariate analysis revealed significant associations with poor OS for age, ECOG performance status, deep brain involvement, number of lesions, and high exosomal ANGPTL2 levels. Multivariate analysis showed that age (*p* = 0.023) and high exosomal ANGPTL2 levels (*p* = 0.007) remained significant independent predictors of poor OS.

**Table 4:** Univariate and multivariate analyses of factors affecting PFS.

Characteristics	Univariate analysis		Multivariate analysis		
	Median months	*p* value	HR	95% CI	*p* value
Age		0.109	1.038	1.008-1.069	0.011
< 60	15.9				
≥ 60	7.9				
Sex		0.695			
Male	15.7				
Female	11.2				
ECOG		0.045	1.264	0.721-2.216	0.414
≤ 1	17.7				
≥ 2	3.5				
LDH		0.618			
Elevated	7.85				
Normal	10.5				
Biopsy type		0.897			
Surgical	7.4				
Stereotactic	14.5				
Deep brain involvement		0.073	0.845	0.410-1.740	0.648
No	19.4				
Yes	6.3				
No. of lesions		0.027	1.837	1.038-3.253	0.037
1	19.4				
≥ 2	3.45				
CSF protein		0.066			
Elevated	4.2				
Normal	16.1				
CSF cell count		0.574			
Elevated	13.95				
Normal	10.50				
ANGPTL2		0.003	2.870	1.615-5.101	< 0.0001
High-level group	3.5				
Low-level group	24.7				

HR, hazard ratio; 95% CI, 95% confidence interval; ECOG, Eastern Cooperative Oncology Group; LDH, lactate dehydrogenase; CSF, cerebrospinal fluid.

**Table 5:** Univariate and multivariate analyses of factors affecting OS.

Characteristics	Univariate analysis		Multivariate analysis		
	Median months	*p* value	HR	95% CI	*p* value
Age		0.049	1.048	1.006-1.090	0.023
< 60	NR				
≥ 60	30.5				
Sex		0.958			
Male	51				
Female	NR				
ECOG		0.028	1.233	0.554-2.741	0.608
≤ 1	NR				
≥ 2	24.4				
LDH		0.604			
Elevated	25.7				
Normal	NR				
Biopsy type		0.799			
Surgical	NR				
Stereotactic	NR				
Deep brain involvement		0.035	1.288	0.452-3.672	0.635
No	NR				
Yes	30.5				
No. of lesions		0.038	1.656	0.764-3.593	0.202
1	NR				
≥ 2	28.4				
CSF protein		0.301			
Elevated	30.5				
Normal	NR				
CSF cell count		0.366			
Elevated	26				
Normal	NR				
ANGPTL2		0.002	2.867	1.332-6.169	0.007
High-level group	28.4				
Low-level group	NR				

HR, hazard ratio; 95% CI, 95% confidence interval; ECOG, Eastern Cooperative Oncology Group; LDH, lactate dehydrogenase; CSF, cerebrospinal fluid.

## Discussion

PCNSL is a highly aggressive malignancy confined to the CNS, with a poor prognosis and limited therapeutic options (4, 20). The BBB poses significant clinical challenges by impeding effective drug delivery and limiting the diagnostic and prognostic utility of peripheral biomarkers (21). Consequently, reliable prognostic biomarkers for patient stratification are critically needed to guide personalized therapeutic strategies and improve clinical outcomes.

In this study, we identified elevated levels of exosomal ANGPTL2 in CSF as a novel prognostic biomarker in PCNSL. High CSF exosomal ANGPTL2 levels independently predicted significantly shorter PFS and OS, providing a quantifiable molecular metric beyond conventional clinical parameters such as age, ECOG performance status, and number of lesions.

Our approach represents an innovative application of liquid biopsy in PCNSL by focusing on CSF-derived exosomal cargo. Unlike peripheral blood, CSF directly interfaces with the CNS tumor microenvironment, bypassing BBB restrictions and providing molecular insights into PCNSL pathobiology (22, 23). The identification of exosomal ANGPTL2 as a prognostic indicator underscores the value of leveraging CSF-derived exosomes to elucidate tumor–host interactions that drive disease aggressiveness.

Current prognostic scoring systems for PCNSL, including the International Extranodal Lymphoma Study Group (IELSG) score (24) and the Memorial Sloan Kettering Cancer Center (MSKCC) score (25), rely heavily on clinical and radiographic variables. While useful in routine practice, these models lack direct molecular biomarkers. Critically, our multivariate analyses strongly demonstrated that CSF exosomal ANGPTL2 levels provided independent prognostic value, exhibiting superior discriminative power compared with established clinical factors and enabling more precise risk stratification of patients.

ANGPTL2 has been implicated in tumor progression in other malignancies by promoting chronic inflammation, cell migration, and invasion (12). Although our data establish a robust association between ANGPTL2 expression and poor prognosis in PCNSL patients, mechanistic studies are needed to determine whether ANGPTL2 directly drives malignant phenotypes, including proliferation, migration, and invasion (13, 14, 26). Future studies using loss-of-function and gain-of-function models in PCNSL cell lines will be essential to elucidate these pathways.

This study has several limitations. First, the sample size was relatively modest, reflecting the rarity of PCNSL, which may limit statistical power and generalizability. Second, we analyzed only clinical features and did not include histopathological or molecular tumor characteristics, potentially omitting additional prognostic variables. Third, CSF exosomal ANGPTL2 was quantified using a volume-based normalization approach, which, while practical and clinically interpretable, may introduce variability due to differences in exosome yield between samples. Alternative strategies, such as normalization to total exosomal protein or particle number, remain technically challenging in CSF due to low vesicle abundance and limited sample volume, and require further validation. Fourth, although all participants received an HD-MTX–based backbone, accompanying agents, number of cycles, and consolidation strategies may vary in real-world practice. Such treatment heterogeneity and potential confounding by indication could influence survival outcomes and may partially contribute to the observed association between baseline ANGPTL2 and prognosis. Notably, all CSF samples were obtained before any antitumor therapy, which reduces the likelihood that posttreatment effects directly altered ANGPTL2 measurements; however, residual confounding cannot be excluded. Fifth, because lumbar puncture is not feasible or clinically appropriate for all patients, and survival analyses require reliable follow-up, selection bias related to CSF availability and follow-up completeness may exist. Finally, this study was retrospective and single-center; prospective, multicenter studies are warranted to confirm the clinical utility and to define optimal thresholds for ANGPTL2 measurement.

In summary, our findings suggest that elevated CSF exosomal ANGPTL2 is a promising prognostic biomarker in PCNSL. Quantifying CSF exosomal ANGPTL2 may help identify high-risk patients at diagnosis who could benefit from intensified or novel first-line therapies and enable real-time monitoring of treatment response and resistance. Moreover, ANGPTL2 is a potential therapeutic target. Overall, measuring CSF exosomal ANGPTL2 represents a step toward biologically informed precision medicine in PCNSL, providing both prognostic insight and mechanistic direction for future research.

## Data Availability

The original contributions presented in the study are included in the article/supplementary material. Further inquiries can be directed to the corresponding author.

## References

[1] Louis DN Perry A Wesseling P Brat DJ Cree IA Figarella-Branger D . The 2021 WHO classification of tumors of the central nervous system: a summary. Neuro-Oncol. (2021) 23:1231–51. doi: 10.1093/neuonc/noab106, PMID: 34185076 PMC8328013

[2] Campo E Jaffe ES Cook JR Quintanilla-Martinez L Swerdlow SH Anderson KC . The international consensus classification of mature lymphoid neoplasms: a report from the clinical advisory committee. Blood. (2022) 140:1229–53. doi: 10.1182/blood.2022015851, PMID: 35653592 PMC9479027

[3] Chihara D Fowler NH Oki Y Fanale MA Nastoupil LJ Westin JR . Impact of histologic subtypes and treatment modality among patients with primary central nervous system lymphoma: a SEER database analysis. Oncotarget. (2018) 9:28897–902. doi: 10.18632/oncotarget.25622, PMID: 29988979 PMC6034756

[4] Ferreri AJM Calimeri T Cwynarski K Dietrich J Grommes C Hoang-Xuan K . Primary central nervous system lymphoma. Nat Rev Dis Prim. (2023) 9:29. doi: 10.1038/s41572-023-00439-0, PMID: 37322012 PMC10637780

[5] Kaulen LD Baehring JM . Treatment options for recurrent primary CNS lymphoma. Curr Treat Options Oncol. (2022) 23:1548–65. doi: 10.1007/s11864-022-01016-5, PMID: 36205806

[6] Korfel A Schlegel U . Diagnosis and treatment of primary CNS lymphoma. Nat Rev Neurol. (2013) 9:317–27. doi: 10.1038/nrneurol.2013.83, PMID: 23670107

[7] Baraniskin A Schroers R . Liquid biopsy and other non-invasive diagnostic measures in PCNSL. Cancers. (2021) 13:2665. doi: 10.3390/cancers13112665, PMID: 34071407 PMC8198992

[8] Fox CP Phillips EH Smith J Linton K Gallop-Evans E Hemmaway C . Guidelines for the diagnosis and management of primary central nervous system diffuse large B-cell lymphoma. Br J Haematol. (2019) 184:348–63. doi: 10.1111/bjh.15661, PMID: 30467845

[9] Krylova SV Feng D . The machinery of exosomes: biogenesis, release, and uptake. Int J Mol Sci. (2023) 24:1337. doi: 10.3390/ijms24021337, PMID: 36674857 PMC9865891

[10] Kok VC Yu C-C . Cancer-derived exosomes: their role in cancer biology and biomarker development. Int J Nanomed. (2020) 15:8019–36. doi: 10.2147/ijn.s272378, PMID: 33116515 PMC7585279

[11] Mutter JA Alig SK Esfahani MS Lauer EM Mitschke J Kurtz DM . Circulating tumor DNA profiling for detection, risk stratification, and classification of brain lymphomas. J Clin Oncol. (2023) 41:1684–94. doi: 10.1200/jco.22.00826, PMID: 36542815 PMC10419411

[12] Kadomatsu T Endo M Miyata K Oike Y . Diverse roles of ANGPTL2 in physiology and pathophysiology. Trends Endocrinol Metab. (2014) 25:245–54. doi: 10.1016/j.tem.2014.03.012, PMID: 24746520

[13] Horiguchi H Kadomatsu T Miyata K Terada K Sato M Torigoe D . Stroma-derived ANGPTL2 establishes an anti-tumor microenvironment during intestinal tumorigenesis. Oncogene. (2021) 40:55–67. doi: 10.1038/s41388-020-01505-7, PMID: 33051596

[14] Kadomatsu T Hara C Kurahashi R Horiguchi H Morinaga J Miyata K . ANGPTL2-mediated epigenetic repression of MHC-I in tumor cells accelerates tumor immune evasion. Mol Oncol. (2023) 17:2637–58. doi: 10.1002/1878-0261.13490, PMID: 37452654 PMC10701769

[15] Yumoto S Horiguchi H Kadomatsu T Horino T Sato M Terada K . Host ANGPTL2 establishes an immunosuppressive tumor microenvironment and resistance to immune checkpoint therapy. Cancer Sci. (2024) 115:3846–58. doi: 10.1111/cas.16348, PMID: 39321028 PMC11611770

[16] Thorin-Trescases N Thorin E . Angiopoietin-like-2: a multifaceted protein with physiological and pathophysiological properties. Expert Rev Mol Med. (2014) 16:e17. doi: 10.1017/erm.2014.19, PMID: 25417860

[17] Huang D Sun G Hao X He X Zheng Z Chen C . ANGPTL2-containing small extracellular vesicles from vascular endothelial cells accelerate leukemia progression. J Clin Invest. (2021) 131:e138986. doi: 10.1172/jci138986, PMID: 33108353 PMC7773400

[18] Horiguchi H Kadomatsu T Yumoto S Masuda T Miyata K Yamamura S . Tumor cell-derived ANGPTL2 promotes β-catenin-driven intestinal tumorigenesis. Oncogene. (2022) 41:4028–41. doi: 10.1038/s41388-022-02405-8, PMID: 35831580

[19] Liu X Qin J Nie J Gao R Hu S Sun H . ANGPTL2+cancer-associated fibroblasts and SPP1+macrophages are metastasis accelerators of colorectal cancer. Front Immunol. (2023) 14:1185208. doi: 10.3389/fimmu.2023.1185208, PMID: 37691929 PMC10483401

[20] Hoang-Xuan K Deckert M Ferreri AJM Furtner J Perez-Larraya JG Henriksson R . European Association of Neuro-Oncology (EANO) guidelines for treatment of primary central nervous system lymphoma (PCNSL). Neuro-Oncol. (2022) 25:37–53. doi: 10.1093/neuonc/noac196, PMID: 35953526 PMC9825335

[21] Schmitz N . Treatment of primary CNS lymphoma. Blood. (2015) 125:1360–1. doi: 10.1182/blood-2015-01-622498, PMID: 25721041

[22] Yi Z Qu C Zeng Y Liu Z . Liquid biopsy: early and accurate diagnosis of brain tumor. J Cancer Res Clin Oncol. (2022) 148:2347–73. doi: 10.1007/s00432-022-04011-3, PMID: 35451698 PMC11800921

[23] Trivedi R Bhat KP . Liquid biopsy: creating opportunities in brain space. Br J Cancer. (2023) 129:1727–46. doi: 10.1038/s41416-023-02446-0, PMID: 37752289 PMC10667495

[24] Ferreri AJM Blay J-Y Reni M Pasini F Spina M Ambrosetti A . Prognostic scoring system for primary CNS lymphomas: the international extranodal lymphoma study group experience. J Clin Oncol. (2003) 21:266–72. doi: 10.1200/jco.2003.09.139, PMID: 12525518

[25] Abrey LE Ben-Porat L Panageas KS Yahalom J Berkey B Curran W . Primary central nervous system lymphoma: the memorial sloan-kettering cancer center prognostic model. J Clin Oncol. (2006) 24:5711–5. doi: 10.1200/jco.2006.08.2941, PMID: 17116938

[26] Endo M Nakano M Kadomatsu T Fukuhara S Kuroda H Mikami S . Tumor cell–derived angiopoietin-like protein ANGPTL2 is a critical driver of metastasis. Cancer Res. (2012) 72:1784–94. doi: 10.1158/0008-5472.can-11-3878, PMID: 22345152

